# GSK-LSD1, an LSD1 inhibitor, quashes SARS-CoV-2-triggered cytokine release syndrome in-vitro

**DOI:** 10.1038/s41392-020-00391-5

**Published:** 2020-11-17

**Authors:** Kyung Soo Hong, June Hong Ahn, Jong Geol Jang, Jong Ho Lee, Hong Nam Kim, Dongha Kim, Wonhwa Lee

**Affiliations:** 1 Division of Pulmonology and Allergy, Department of Internal Medicine, College of Medicine, Yeungnam University and Regional Center for Respiratory Diseases, Yeungnam University Medical Center, Daegu, 42415 Republic of Korea; 2 Department of Laboratory Medicine, College of Medicine, Yeungnam University, Yeungnam University Medical Center, Daegu, 42415 Republic of Korea; 3 Brain Science Institute, Korea Institute of Science and Technology (KIST), Seoul, 02792 Republic of Korea; 4 Division of Bio-Medical Science and Technology, KIST School, Korea University of Science and Technology (UST), Seoul, 02792 Republic of Korea; 5 Department of Anatomy, College of Medicine, The Catholic University of Korea, Seoul, 06591 Republic of Korea; 6 Aging Research Center, Korea Research Institute of Bioscience and Biotechnology, Daejeon, 34141 Republic of Korea

**Keywords:** Molecular medicine, Drug development

**Dear Editor,**

Since the COVID-19 demonstrated a remarkable transmission speed and a high mortality risk, COVID-19 is currently declared as a pandemic by the World Health Organization. This is the first time that the WHO has declared a pandemic since H1N1 in 2009. As of 6 September 2020, the total number of COVID-19 patients is 26,763,217 (876,616 deaths), and it continues to rise (https://covid19.who.int/). In spite of the urgent demand for the vaccines and therapeutics, global efforts are primarily focused on the utilization of the existing anti-viral drugs, such as remdesivir, hydroxychloroquine, and dexamethasone, to relieve the symptoms due to the limited development of renovative therapeutics. Therefore, more fundamental solutions are urgently needed to overcome the threat of the COVID-19. The deterioration of the COVID-19 patients with acute respiratory distress syndrome (ARDS) and sepsis are mainly caused by the cytokine storm, the overproduced cytokines by immune cells. Previous studies indicated that the infection does cause the cytokine storm and subsequently ARDS, and may lead to mortality.^1^ Therefore, the suppression of the cytokines and subsequent ARDS is important to increase survival rate through the regulation of cytokine gene expression.^2^ Although NF-κB signaling is known to regulate the cytokine storm, direct targeting of NF-κB is not beneficial since the p65 is readily hyperactivated in COVID-19 patients. Here, we applied a mechanism by which previously reported phosphorylated lysine-specific demethylase 1 (LSD1) stabilizes NF-κB p65 to control the expression of pro-inflammatory cytokine genes.^3^ We found that pro-inflammatory cytokines were significantly reduced when severe COVID-19 patients’ peripheral blood mononuclear cells (PBMCs) were treated in-vitro with Go6976 (inhibitor of LSD1 phosphorylation) or GSK-LSD1 (inhibitor of LSD1 activity). Therefore, this study suggests the GSK-LSD1 as a therapeutic agent for severe COVID-19 patients that are difficult to treat by directly targeting NF-κB.

COVID-19 patients with ARDS and sepsis (severe COVID-19) displayed the uncontrolled NF-κB p65-related cytokine release syndrome (CRS). From 40 blood samples of Normal or severe COVID-19 patients (information in Supplementary Table 1), we evaluated the expression of total LSD1 (Fig. 1a) and phosphorylated-S112 LSD1 (Fig. 1b, c) that regulates nuclear NF-κB p65 stability (Fig. 1d) in isolated PBMCs. The expression of total LSD1 and phosphorylated-S112 LSD1 was increased in PBMCs isolated from severe COVID-19 patients (severe COVID-19 PBMCs) compared to PBMCs isolated from normal individuals (normal PBMCs). Moreover, the LSD1 phosphorylation ratio, defined as the ratio of phosphorylated-S112 LSD1 and total LSD1, was significantly higher in the severe COVID-19 PBMCs than normal PBMCs (Fig. 1a–c). The upregulated LSD1 phosphorylation maintained the stability of nuclear NF-κB p65 and significantly decreased the viability of PBMCs (Fig. 1d, e).

**Fig. 1:**
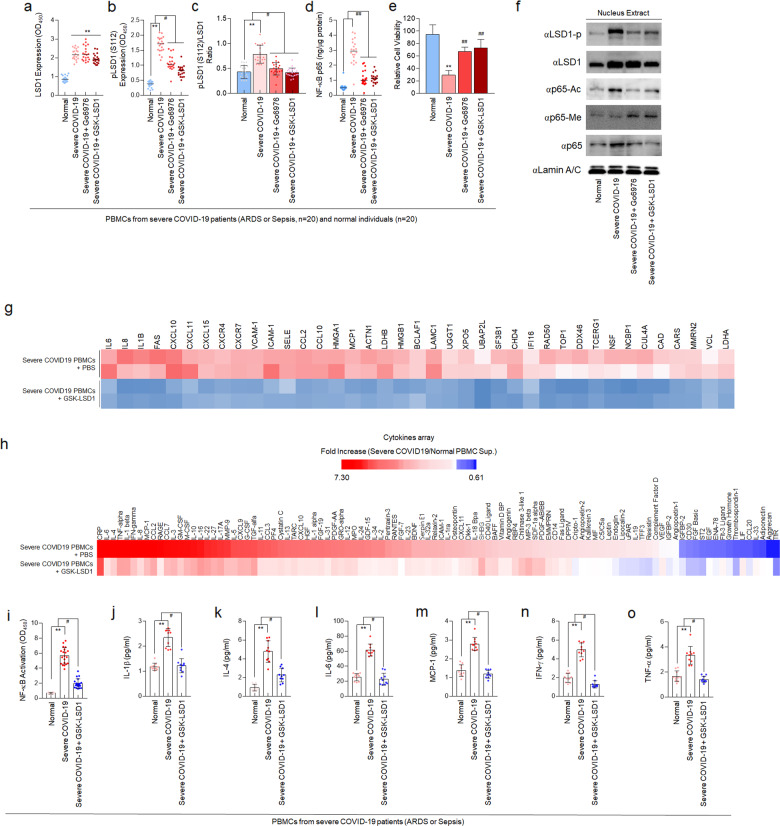
GSK-LSD1 inhibits NF-κB p65-mediated cytokine storm in PBMCs isolated from severe COVID-19 patients. PBMCs were isolated from 20 healthy volunteers and 20 severe COVID-19 patients, respectively. **a**–**c** Level of total LSD1 (**a**), phosphorylated-S112 LSD1 (**b**), and phosphorylation ratio of LSD1 in normal and severe COVID-19 PBMCs (**c**). **d** NF-κB p65 activity in normal and severe COVID-19 PBMCs. **e** Viability of PBMCs isolated from severe COVID-19 patients. **f** Immunoblot of phosphorylated-S112 LSD1, LSD1, acetylated K314/315-, monomethylated K314/315-NF-κB p65, and nucleus NF-κB p65 was shown in Go6976 or GSK-LSD1-treated PBMCs from normal and severe COVID-19 PBMCs. **g** Heat map of inflammation-related genes in PBMCs from severe COVID-19 patients after GSK-LSD1 treatment (*n* = 3). **h** Cytokines profiling of pharmacological effect of GSK-LSD1 for preventing cytokine storm. **i** Binding activity of NF‑κB p65 in normal and severe COVID-19 PBMCs. **j**–**o** Plasma cytokines levels in normal and GSK-LSD1-treated severe COVID-19 PBMCs. Data are reported as mean ± SEM. Significance was set at **P < 0.01 vs. normal; ^#^*P* < 0.05, ^##^*P* < 0.01 vs. severe COVID-19

A recent news from Oxford University in the UK reported that the anti-inflammatory drug dexamethasone improved the survival of severe COVID-19 patients.^4^ Previous studies also indicated that the dexamethasone inhibited the expression of pro-inflammatory cytokines by inhibiting the JAK-STAT pathway.^5^ Furthermore, our previous studies found that Go6976, an inhibitor of PKCα that phosphorylates LSD1, and GSK-LSD1, which directly inhibits activation of LSD1, could improve survival rate in the septic mouse model by inhibiting the PKCα-LSD1-NF-κB pathway.^3^ In this regard, we treated the PBMCs isolated from severe COVID-19 patients with Go6976/GSK-LSD1. Interestingly, the total level of LSD1 expression remained unchanged, but the phosphorylated-S112 LSD1 was reduced, and thus the LSD1 phosphorylation ratio was also significantly reduced (Fig. 1a–c). The Go6976 and GSK-LSD1 also decreased the expression of nuclear NF-κB p65 and ameliorated the cell viability of severe COVID-19 PBMCs (Fig. 1e). Therefore, we confirmed the Go6976 or GSK-LSD1 as potential therapeutics for severe COVID-19 patients, which can suppress the demethylation of NF-κB p65 by LSD1 and the expression of pro-inflammatory cytokine genes in vitro. Similar to the results of previous study,^3^ in severe COVID-19 PBMCs, as in other infectious diseases, NF-κB p65 is stabilized through demethylation of LSD1, along with increased acetylation of NF-κB p65, an activated form. Whereas, when severe COVID-19 PBMCs were treated with Go6976 and GSK-LSD1, demethylation of NF-κB p65 by LSD1 was suppressed, and methylation was maintained. As a result, the stability of NF-κB p65 was reduced, and the acetylation of NF-κB p65 was also significantly reduced (Fig. 1f).

Because the drug is currently undergoing phase I clinical assessments an agent for cancer therapy (ClinicalTrials.gov),^6^ we intensively evaluated whether GSK-LSD1 has an inhibitory effect on cytokine production. It provides important preclinical information on the shortening of the drug development process through drug repositioning. GSK-LSD1 attenuated the expression of NF-κB-dependent pro-inflammatory cytokine genes in severe COVID-19 PBMCs (Fig. 1g). In addition, as a result of confirming cytokine profiling produced from severe COVID-19 PBMCs (Fig. 1h), it was verified that the production of cytokines was attenuated by significantly suppressing the activity of NF-κB p65 (Fig. 1i). After the treatment with GSK-LSD1, quantitative analysis of secreted cytokines demonstrated a markedly decrease in the selectively upregulated levels of pro-inflammatory cytokines such as IL-1β, IL-4, IL-6, MCP-1, IFN-γ, and TNF-α (Fig. 1j–o).

In previous study, we found that inhibitors of the PKCα-LSD1-NF-κB signaling pathway,^3^ Go6976 and GSK-LSD1, that inhibited the expression of several cytokines at once in severe COVID-19 PBMCs. These results may overcome the limitations of existing single cytokine inhibitor for improved suppression of ARDS and sepsis in severe COVID-19 patients. In addition, GSK-LSD1, which has already been undergoing clinical approval as cancer therapy,^6^ can be applied directly to severe COVID-19 patients, thereby saving time and cost, and be used as a therapeutic agent that can decrease mortality of severe COVID-19. The best treatment strategy to reduce the COVID-19 severity is to co-administer anti-viral and anti-inflammatory drugs that suppress viral infection and severe inflammatory responses.

## Supplementary information

SUPPLEMENTAL MATERIAL

## Data Availability

The raw/processed data required to reproduce these findings cannot be shared at this time due to legal or ethical reasons.
