# Supplementary material for: GSK-LSD1, an LSD1 inhibitor, quashes SARS-CoV-2-triggered cytokine release syndrome in-vitro
Source: Signal Transduct Target Ther. 2020 Nov 17;5:267. doi: 10.1038/s41392-020-00391-5 (PMC7670835; doi:10.1038/s41392-020-00391-5)
Supplement: Supplementary file 1 — SUPPLEMENTAL MATERIAL [file 41392_2020_391_MOESM1_ESM.docx]

Supplementary Materials for

**GSK-LSD1, an LSD1 Inhibitor, Quashes SARS-CoV-2-triggered Cytokine Release Syndrome *in-vitro***

Kyung Soo Hong ^#1^, June Hong Ahn ^#1^, Jong Geol Jang ^#1^, Jong Ho Lee ^2^, Hong Nam Kim *^3,4^, Dongha Kim *^5^, Wonhwa Lee *^6^

^#^These authors contributed equally: Kyung Soo Hong, June Hong Ahn, Jong Geol Jang

^*^Co-corresponding author: Hong Nam Kim ([hongnam.kim@kist.re.kr](mailto:hongnam.kim@kist.re.kr)), Dongha Kim ([hidongha@catholic.ac.kr](mailto:hidongha@catholic.ac.kr)), and Wonhwa Lee ([wonhwalee@kribb.re.kr](mailto:wonhwalee@kribb.re.kr))

**Supplementary information include:**

Materials and Methods

Supplemental table 1.

Additional References

**Materials and Methods**

**COVID-19 patient sample**

Whole blood was collected from patients admitted at Yeungnam University Medical Center after they were diagnosed with the SARS-CoV-2 infection at a public health center in Daegu, Republic of Korea. We defined COVID-19 patient with sepsis or ARDS (acute respiratory distress syndrome) as severe COVID-19 patients based on criteria provided by the Sepsis Consensus Conference Committee. [1] In addition, the severe COVID-19 patients were in a state of difficulty in self-breathing and progress mechanical ventilator and oxygen therapy in intensive care unit (ICU). Eligible patients met the following criteria: age, over 18 years; and an acute onset medical condition with as at least one of the following criteria: fever (tympanic temperature ≥ 38°C at the nurse triage), suspected systemic infection, two or more systemic inflammatory response syndrome (SIRS) criteria, and hypotension (systolic blood pressure < 90 mmHg), and/or shock. Healthy volunteers were used as controls. Clinical data were collected for all the patients. Plasma samples were prepared by centrifugation at 2000 ×*g* for 5 min within 12 h after whole blood collection. The human study protocol was approved by the Institutional Review Board of Yeungnam University Hospital at Daegu in Korea (YUH 2020-03-057 and 2020-05-031-001).

**PBMC isolation and culture**

Samples from healthy, SARS-CoV-2 pneumonia patients, and discharged patients were obtained from Yeungnam University Medical Center. The relevant local Institutional Review Boards and Ethics Committees approved the study. Heparinized blood samples were used freshly within 4 h, and peripheral blood mononuclear cells (PBMCs) were separated from blood using Ficoll–Hypaquek or NycoPrepk according to the manufacturer’s recommendations. Following this, more refined PBMCs were obtained via MACSprep™ PBMC Isolation Kit and cultured in RPMI-1640 with 1 mM Sodium pyruvate, 2 mM L-glutamine, 4.5 mg/L glucose, 10mM HEPES, and 2 mg/L sodium bicarbonate. To verify the effect of Go6976 or GSK-LSD1 on the suppression of cytokine secretion and NF-κB activation by LSD1 inactivation, PBMCs isolated from SARS-CoV-2 patients were incubated with the Go6976 (20 μg/ml) or GSK-LSD1 (10 ng/ml) for 6 hours.

**Phospho-LSD1 (S112) and Total LSD1 ELISA**

Phospho-LSD1 (S112) and Total LSD1 were measured using Human and Mouse Phospho-LSD1 (S112) and Total LSD1 ELISA kit (PEL-LSD1-S112-T-1, Raybiotech, USA) according to the manufacturer’s protocols. This assay semi-quantitatively measures phosphorylated LSD1 (Ser112) and Total LSD1 in the lysate samples of the severe COVID-19 patients’ PBMCs.

**NF-kB activity assay**

Preparation of the nuclear extracts and the TransAM assays were performed as previously described.[2] The activity of individual NF-κB subunits was determined using an ELISA-based NF-κB Family Transcription Factor Assay Kit (43296; Active Motif, Carlsbad, CA, USA). Briefly, nuclear extracts (2 µg) were incubated in a 96-well plate, which was coated with NF-κB consensus oligonucleotides. The captured complexes were incubated with specific NF-κB primary Abs and subsequently detected using HRP-conjugated secondary Abs included with the kit. Finally, the optical density (OD) at 450 nm was measured using a Tecan Spark microplate reader (Tecan, Austria GmbH, Austria).

**WST-1 cell proliferation assay**

10 μL per well of WST-1 reagent were added in Go6976 or GSK-LSD-treated severe COVID-19 patients’ PBMC and incubated at 37 °C with 5% CO_2_. At indicated time points, measurements of absorbance were taken at 480 nm and 600 nm (background) on Tecan Spark microplate reader (Tecan, Austria GmbH, Austria).

**Western blot**

NF-κB and LSD1 in nucleus extraction of Go6976- or GSK-LSD-treated severe COVID-19 patients’ PBMC were detected by immunoblotting. After SDS-PAGE, we performed an immunoblotting assay with each antibody. Anti-phospho LSD1 (Ser 112) antibody (ABE1462, Sigma-Aldrich, USA), Anti-LSD1 antibody (ABE365, Sigma-Aldrich, USA), anti-NF-kB p65 acetyl K310 antibody (ab19870, Abcam, United Kingdom), anti-NF-κB p65 Methyl K310 antibody (13188, Cell Signaling Technology, USA), anti-NF-κB p65 antibody (8242, Cell Signaling Technology, USA), and anti-Lamin A/C antibody (4777, Cell Signaling Technology, USA)

**RNA analysis**

RNA from PBS-treated severe COVID-19 patients’ PBMC or GSK-LSD1-treated severe COVID-19 patients’ PBMC was extracted using RNeasy mini-kit (Qiagen Venlo, Netherlands) according to the manufacturer’s protocols. RNA-seq libraries were prepared using the TruSeq RNA Sample Prep kit v2 (Illumina, USA) according to the manufacturer’s protocols. RNA-seq libraries were pair-end sequenced on an Illumina Hi-seq 3000/4000 SBS kit v3 (MACROGEN Inc., Korea). All RNA-seq data were mapped using the Tophat package [3] against Affymetrix Human Gene 2.0 ST arrays (902136). Remaining mRNA was used for qPCR analysis. Fold-change was determined using the R package limma, and P-values were adjusted using Benjamini-Hochberg (BH) procedure. The array results are available in the Gene Expression Omnibus (GEO) database of NCBI (Accession code: GSE101126).

**Cytokine profiling and ELISAs**

After incubating the PBMCs of severe COVID-19 patients with or without GSK-LSD1, secreted cytokines in cultured media were measured with a supernatant containing unbound cytokines and a pellet containing cytokines bound to the substance. Cytokines were processed as indicated in the Human XL Cytokine Array Kit (R&D Systems, Minneapolis, MN, USA). Developed films were scanned, the obtained images were analyzed using ImageJ version 1.43. Levels of inflammatory cytokines IL-1β, IL 4, IL-6, IFN-γ, and TNF-α were measured using human ELISA kits (Quantikine ELISA, R&D Systems, Minneapolis, MN, USA) according to the manufacturer’s protocols.

**Statistical analysis**

All experiments were independently performed at least three times. Statistically significant differences were determined using the unpaired t-test. GraphPad Prism software was used for statistical analyses. Data are reported as mean ± SEM with significance set at *P* < 0.05. *P*-values and detailed information for each experiment are provided in the figure legends.

**Supplemental table 1.**

**Table S1. Baseline characteristics and clinical outcomes of severe COVID-19 patients in admitted to Yeungnam University Hospital**

|  | Normal  (n=20) | Severe COVID-19  (n=20) |
| --- | --- | --- |
| **Characteristics** |  |  |
| Age, y | 35.4 ± 17.4 | 74.6 ± 12.8 |
| Sex |  |  |
| Men | 10(50) | 13 (65) |
| Women | 10(50) | 7 (35) |
| Onset of symptom to hospital admission  (days, mean) |  | 3.5 ± 3.6 |
| **Symptoms at admission** |  |  |
| Fever | 0 (0) | 16 (80) |
| Cough | 0 (0) | 17 (85) |
| Sputum | 0 (0) | 13 (65) |
| Rhinorrhea | 0 (0) | 0 (0) |
| Sore throat | 0 (0) | 0 (0) |
| Dyspnea | 0 (0) | 15 (75) |
| Chest pain | 0 (0) | 10 (50) |
| Headache | 0 (0) | 0 (0) |
| Confusion | 0 (0) | 0 (0) |
| Myalgia | 0 (0) | 6 (60) |
| Fatigue | 0 (0) | 11 (55) |
| Anorexia | 0 (0) | 0 (0) |
| Nausea or vomiting | 0 (0) | 0 (0) |
| Diarrhea | 0 (0) | 0 (0) |
| **Comorbidities** |  |  |
| Cardiovascular disease | 0 (0) | 0 (0) |
| Cerebrovascular disease | 0 (0) | 0 (0)) |
| ^*^Chronic lung disease | 0 (0) | 0 (0) |
| Dementia | 0 (0) | 0 (0) |
| Diabetes mellitus | 0 (0) | 3 (15) |
| Hypertension | 0 (0) | 5 (25) |
| Liver disease | 0 (0) | 0 (0) |
| Malignancy | 0 (0) | 1 (5) |
| Parkinson’s disease | 0 (0) | 1 (5) |
| **Clinical outcomes** |  |  |
| Remained in hospital | 0 (0) | 3 (15) |
| Discharged | 0 (0) | 0 (0) |
| Died | 0 (0) | 17 (85) |
| Transferred for intensive care | 0 (0) | 20 (100) |

Data are presented as mean ± SD (range) or number (percentage).

* Chronic lung disease includes COPD, asthma, bronchiectasis, and interstitial lung disease.

ICU = intensive care unit, SARS-CoV-2 = severe acute respiratory syndrome coronavirus 2

**Additional References**

1. Singer, M. *et al.* The third international consensus definitions for sepsis and septic shock (Sepsis-3). *JAMA* **315**, 801-810 (2016).

2. Xu, J., Zhou, P., Wang, W., Sun, A. & Guo, F. RelB, together with RelA, sustains cell survival and confers proteasome inhibitor sensitivity of chronic lymphocytic leukemia cells from bone marrow. *J Mol Med* **92**, 77-92 (2014).

3. Kim, D. *et al.* TopHat2: accurate alignment of transcriptomes in the presence of insertions, deletions and gene fusions. *Genome Biol* **14**, doi:10.1186/gb-2013-1114-1184-r1136 (2013).
